# Caffeine may disrupt the impact of real-time drowsiness on cognitive performance: a double-blind, placebo-controlled small-sample study

**DOI:** 10.1038/s41598-021-83504-6

**Published:** 2021-02-17

**Authors:** E. Aidman, M. Balin, K. Johnson, S. Jackson, G. M. Paech, M. Pajcin, C. Yates, E. Mitchelson, G. H. Kamimori, J. Fidock, C. Della Vedova, S. Banks

**Affiliations:** 1 Defence Science and Technology Group, Edinburgh, 5111 Australia; 2 Behaviour-Brain-Body Research Group, Justice and Society, University of South Australia, Adelaide, Australia; 3 Clinical and Health Sciences, University of South Australia, Adelaide, Australia; 4 Behavioral Biology Branch, Center for Military Psychiatry and Neuroscience Research, Walter Reed Army Institute of Research, Silver Spring, USA; 5 School of Psychology, University of Sydney, Sydney, Australia; 6 School of Biomedical Sciences & Pharmacy, University of Newcastle, Callaghan, Australia

**Keywords:** Physiology, Human behaviour

## Abstract

Caffeine is widely used to promote alertness and cognitive performance under challenging conditions, such as sleep loss. Non-digestive modes of delivery typically reduce variability of its effect. In a placebo-controlled, 50-h total sleep deprivation (TSD) protocol we administered four 200 mg doses of caffeine-infused chewing-gum during night-time circadian trough and monitored participants' drowsiness during task performance with infra-red oculography. In addition to the expected reduction of sleepiness, caffeine was found to disrupt its degrading impact on performance errors in tasks ranging from standard cognitive tests to simulated driving. Real-time drowsiness data showed that caffeine produced only a modest reduction in sleepiness (compared to our placebo group) but substantial performance gains in vigilance and procedural decisions, that were largely independent of the actual alertness dynamics achieved. The magnitude of this disrupting effect was greater for more complex cognitive tasks.

## Introduction

Sustained operations require operators to maintain their vigilance and task performance in the face of growing fatigue—both physical and cognitive^1–4^. There is a growing demand for effective countermeasures under performance-degrading conditions, such as increasing sleep deficit^5,6^. Caffeine is one of the safest and most commonly used fatigue countermeasures^7,8^. Extensive research on the benefits of caffeine as a mild stimulant, suggests that its performance-protecting and enhancing effects are linked to its capacity to promote alertness^4,5,9^. Its overall utility in the field is influenced by the available mode of delivery and dosage precision^10–12^. Drug formulation has been shown to influence its pharmacokinetics after oral administration, and its overall effectiveness in maintaining alertness, with chewing gum formulations enabling superior rates of absorption than pharmaceutical grade caffeine (tablet or capsule) while producing comparable amounts of caffeine to the systemic circulation^19^. Our study examined the effects of chewing gum-administered caffeine on the well-established relationship between drowsiness and cognitive performance, under conditions of accumulating sleep loss^13^. We have previously found that caffeine administered via chewing gum and strategically timed to circadian trough periods during sleep deprivation, was able to reduce subjective fatigue, mental exhaustion and irritability^14^ and rescue the declining performance on a simulated driving task^15^. It also improved performance on standard tests of vigilance and working memory^16^ while having no substantial impact on the recovery sleep^17^, glucose metabolism and feelings of hunger^14^. Overall performance deficit in cognitive test performance was associated with levels of salivary alpha-amylase^18^, while the fluctuating dynamics of driving performance were linked to real-time eye-blink-derived drowsiness estimates^13^, indicating substantial differences in time resolution among potential biomarkers of performance under sleep deprivation. The latter study^13^ found an unexpected reduction in the magnitude of real-time covariation between the eye-blink drowsiness marker and driving performance in our caffeine group (compared to controls). This finding suggested that, in addition to reducing drowsiness overall, caffeine may disrupt the impact of its moment-to-moment dynamics on driving performance, e.g., making driving errors less influenced by real-time fluctuations in drowsiness. If confirmed, this finding may have substantial implications, pointing to an additional, yet to be examined, mechanism behind the performance enhancement effect of caffeine. Our current study aimed at providing such conceptual confirmation by examining the connection between real-time drowsiness and previously unpublished data (collected in the same experiment) on cognitive task performance of varying complexity, from simple reaction time to procedural decisions and response inhibition. We hypothesised that caffeine may cause a similar dissociation between real-time drowsiness and task performance observed in driving^13^ and the magnitude of this dissociation may depend on task complexity. In other words, we expected that (a) caffeine would weaken the positive associations between real-time drowsiness and response time and errors in cognitive tasks, and (b) that this weakening effect would be stronger for more complex tasks (e.g., a Go-NoGo task) compared to simpler tasks (e.g., a choice or simple reaction task).

Twenty-four adults (12 male) aged 18–31 years were randomly allocated into either placebo or caffeine group and participated in a 50-h total sleep deprivation (TSD) protocol. The caffeine group consumed four doses of caffeine via gum pellets (200 mg per dose) every 2 h (0100, 0300, 0500, 0700) on the first and second nights of sleep deprivation. The placebo group consumed non-caffeinated chewing gum that was identical in appearance and flavour, at the same time points. All participants were instructed to chew the pellets for a minimum of 5 min, as prior research shows that 85% of the dose is released within 5 min of chewing^19^. Following a 10-h sleep opportunity (2200–0800), participants were constantly monitored and remained awake for 50 h. Every 3 h participants drove for 40 min continuously in a medium-fidelity driving simulator. During the driving task, real-time drowsiness was monitored for n = 5 caffeine and n = 6 placebo group participants who did not wear prescription spectacles, and their data are included in the present analysis. A spectacle-worn infra-red oculography monitor (Optalert, Melbourne, Australia) quantified drowsiness levels in the form of the Johns Drowsiness Scale (JDS) scores^20–22^. Immediately before and after each driving task, participants’ cognitive performance was measured with a brief, 3-min Psychomotor Vigilance Test (PVT)^23–25^. Full details of this protocol have been published elsewhere^13^. In addition to the previously published data, three sub-tests from the Defense Automated Neuropsychological Assessment (DANA) battery^26–28^ were added for the current analysis—the simple reaction, the two-choice procedural decision and the Go-NoGo tasks (see “Methods” for subtest descriptions). These tasks extended the range of cognitive task complexity over the previously published analyses and were added in order to test our second hypothesis that task complexity may modulate the strength of caffeine-induced dissociation between real-time drowsiness and cognitive performance.

JDS data for one participant was unusable due to equipment malfunctioning. To compensate for the resulting small sample size (n = 10) we exploited the multiple repeat measurements in our protocol (a minimum of 15 per participant) and mixed-effects modelling which takes advantage of these repeat measurements and is robust to violations of normality typical in small samples. Individual JDS scores averaged over the 5-min window immediately preceding the cognitive testing, formed the drowsiness predictor of cognitive performance.

## Results

Prior to undergoing TSD, participants were given a 10 h baseline sleep opportunity (monitored via actigraphy and sleep diary) with no differences observed in total sleep time between placebo (8.7 ± 0.49 h) and caffeine (8.9 ± 0.52 h) groups. The groups reported no differences in prior caffeine consumption (1.52 ± 0.71 cups per day in the caffeine group and 1.51 ± 0.7 in placebo) or habitual sleep duration (7.3 ± 0.95 h in the caffeine group and 7.5 ± 0.85 h in placebo).

Cognitive performance declined as drowsiness increased from the first driving task to the last, in a pattern driven by homeostatic drive for sleep (time since wake). Within 1 h of the first caffeinated gum dose, the caffeine group got significantly less drowsy than placebo controls and, as we reported earlier^13^, (Fig. 2), they continued to track about 2 points below the placebo group on the 10-point JDS scale. However, they also reached drowsiness levels that are considered high-risk^22^, with the maximum raw JDS score of 6.8 observed at the end of the protocol (5.4 when averaged over 5 min preceding the cognitive testing). This indicates that caffeine did not protect the participants from getting seriously drowsy. By comparison, our placebo group reached a maximum raw JDS score of 8.2 (7.9 when averaged), only 1.4 higher than the caffeine group (1.6 when averaged), on the 10-point JDS scale.

**Figure 1:**
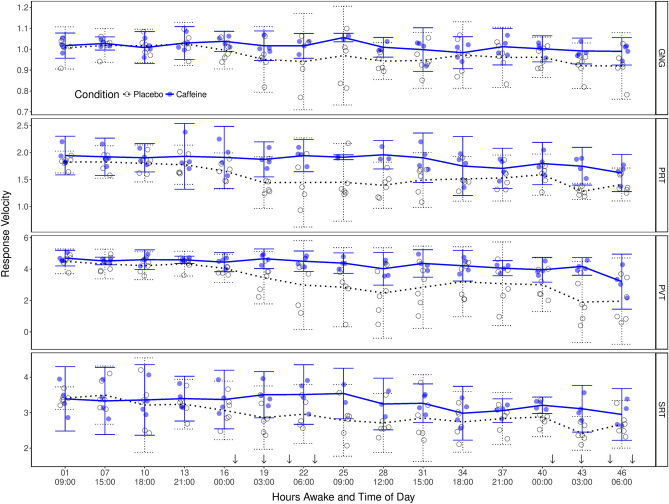
The time course of response speed (left y-axis) on vigilance (PVT and SRT panels denoted on the right), procedural decision making (PRT panel) and response inhibition tasks (GNG panel) for the caffeine (black filled circle, *n* = 4) and placebo (black open circle, *n* = 6) groups across our 50-h TSD protocol. Discrete values represent Response Velocities (per second) measured in PVT, SRT, PRT and GNG tasks following each simulated drive. The x-axes represent hours of continuous wakefulness (top) and time of day in a 24-h format (bottom). Arrows (↓) indicate the time of caffeine administration. Error bars represent 95% CIs.

**Figure 2:**
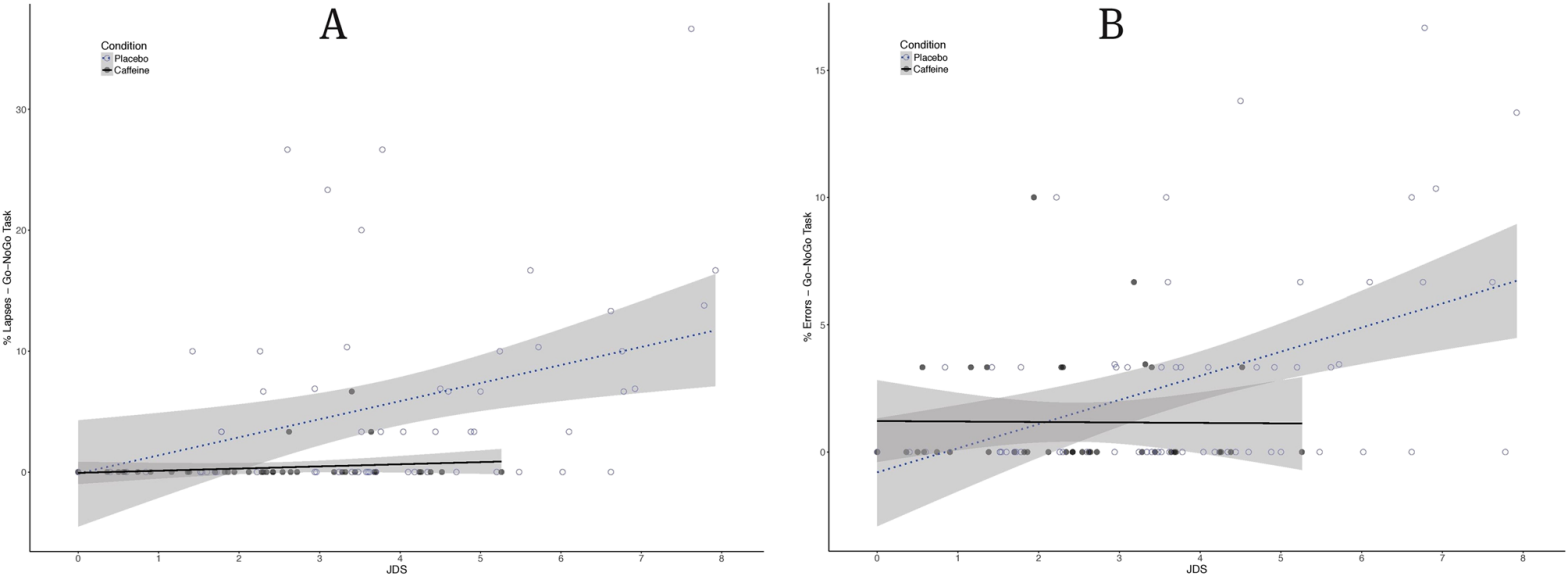
Performance on the Go-NoGo task (GNG) as a function of drowsiness (JDS scores averaged over 5 min prior to the task) for the caffeine (black filled circle, *n* = 4) and placebo (black open circle, *n* = 6) groups across our 50-h TSD protocol. (**A**) Percentage of Response Lapses (omission errors). (**B**) Percentage of commission errors (false alarms). Shaded areas represent 95% CIs over regression lines.

**Figure 3:**
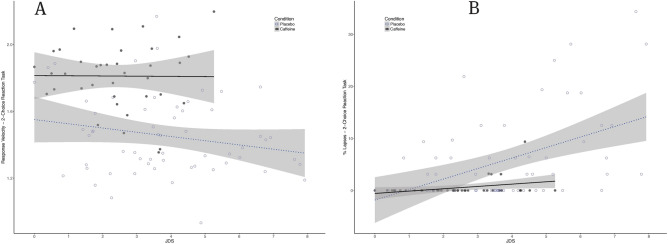
Performance on the 2-Choice Reaction task (PRT) as a function of drowsiness (JDS scores averaged over 5 min prior to the task) for the caffeine (black filled circle, *n* = 4) and placebo (black open circle, *n* = 6) groups across our 50-h TSD protocol. (**A**) Response velocities (s^-1^). (**B**) Percentage of Response Lapses. Shaded areas represent 95% CIs over regression lines.

**Figure 4:**
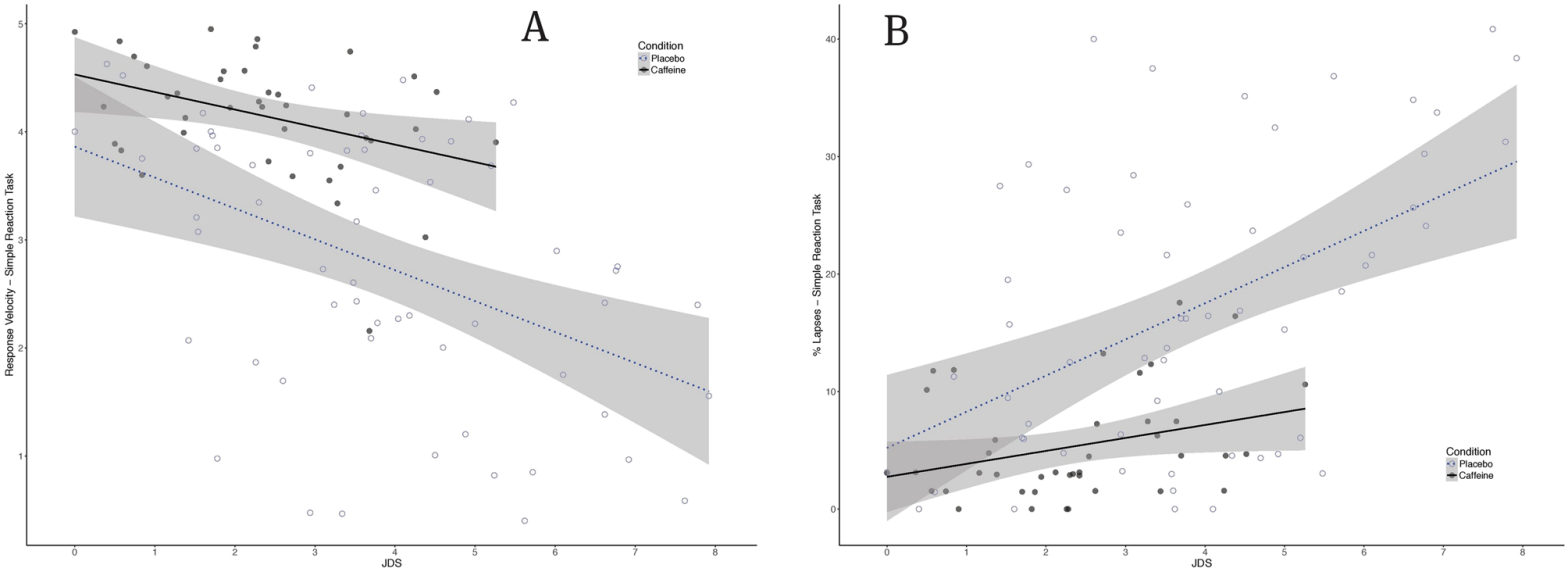
Performance on the Simple Reaction task (3-min PVT) as a function of drowsiness (JDS scores averaged over 5 min prior to the PVT task) for the caffeine (black filled circle, *n* = 4) and placebo (black open circle, *n* = 6) groups across our 50-h TSD protocol. (**A**) Response velocities (fastest 10% reactions, s^−1^). (**B**) Percentage of Response Lapses (reaction time > 355 ms). Shaded areas represent 95% CIs over regression lines.

Figure 1 shows the chronological pattern of cognitive performance across the two groups. The differences between the caffeine and placebo groups did not reach statistical significance even after 19 h of continuous wakefulness and during circadian troughs. Significant differences emerged when time (hours awake) was replaced with JDS scores on the x-axis (see Figs. 2, 3 and 4). Table 1 shows significant main effects of drowsiness on performance in all cognitive tasks: JDS scores preceding the cognitive tasks were associated with response velocities and lapses in both simple reaction tasks: post-drive PVT (Satterthwaite approximated *t* (85.17) = − 4.63, *p* < 0.001 for response times and *t* (94.9) = 4.58, *p* < 0.001 for lapses) and SRT (*t* (91.29) = − 2.05, *p* = 0.04 for response times and *t* (59.9) = 3.88, *p* < 0.001 for lapses). Similar patterns were observed in PRT (two-choice procedural decision time *t* (91.78) = − 4.34, *p* < 0.001) and *t* (39.22) = 4.58, *p* < 0.001 for lapses), as well as in GNG task errors (*t* (71.53) = 2.34, *p* = 0.02) and lapses (*t* (75.88) = 2.91, *p* = 0.005).

**Table 1:** Effects of drowsiness (JDS), caffeine (*n* = 4 vs. placebo, *n* = 6) and their interaction on task performance in simple reaction (PVT and SRT), procedural reaction (PRT) and response inhibition (Go-NoGo) tasks.

	Main Effect: Drowsiness (JDS)			Main effect: caffeine versus placebo			JDS x caffeine interaction effect		
	t	df	*p*	t	df	*p*	t	df	*p*
**PVT**									
Mean RT	− 4.63	85.17	< 0.001***	2.75	94.93	0.02*	1.36	94.93	0.18
False starts	0.81	74.48	0.42	2.00	93.42	0.07	0.08	93.42	0.94
Lapses	4.58	94.99	< 0.001***	− 2.06	93.13	0.07	− 1.76	93.13	0.08
**SRT**									
Mean RT	− 2.05	91.29	0.04*	1.71	91.08	0.12	− 0.78	91.08	0.43
False starts	3.34	92.00	0.001**	− 0.87	92.00	0.39	− 1.41	92.00	0.16
Lapses	3.88	59.89	< 0.001***	− 1.68	86.83	0.13	− 1.66	86.83	0.10
**PRT**									
Mean RT	− 4.34	91.78	< 0.001***	2.46	90.56	0.04*	1.66	90.56	0.10
False starts	0.04	92.00	0.97	− 0.25	92.00	0.80	0.47	92.00	0.64
Lapses	4.58	39.22	< 0.001***	− 2.27	72.18	0.05*	− 1.82	72.18	0.07
Errors	2.63	53.21	0.01*	− 1.84	83.48	0.10	− 1.45	83.48	0.15
**Go-NoGo**									
Mean RT	− 1.92	88.29	0.06	1.55	91.85	0.16	0.41	91.85	0.69
False starts	1.92	35.60	0.06	− 0.66	68.81	0.52	− 0.67	68.81	0.50
Lapses	2.91	75.88	0.005**	− 1.87	90.94	0.09	− 1.40	90.94	0.16
Commission errors	2.34	71.53	0.02*	− 1.33	90.44	0.22	− 2.04	90.44	0.04*

Notes: Non-integer *df* values are the result of effect size estimation with Satterthwaite approximated *t* statistics derived from mixed linear modelling analyses.

*RT* reaction time, *PVT* only post-driving PVT data are presented that could be matched to preceding JDS scores.

^*^p < 0.05; **p < 0.01; ***p < 0.001.

Caffeine significantly improved response time in PVT and PRT tasks, reduced lapses in the latter but did not affect SRT or GNG performance. Importantly, the drowsiness by caffeine interaction effects (last column in Table 1) show that caffeine moderated the effect of drowsiness on cognitive performance.

In particular, as can be seen in Fig. 2 and Table 1, a significant dissociation between drowsiness and response inhibition performance was observed (*t* (90.94) = − 2.042, *p* = 0.04, for JDS x caffeine interaction effect on Go-NoGo commission errors). The same interaction was nearing significance for procedural decision performance (*t* (72.18) = − 1.82, *p* = 0.07 for the lapses in the two-choice reaction task—see Fig. 3) and vigilance performance (PVT lapses: *t* (93.13) = − 1.76 , *p* = 0.08, see Fig. 4) but disappeared almost completely for Simple Reaction times (*t* (90.67) = − 0.79, *p* = 0.434).

## Discussion

The current study continues our inquiry into the effects of caffeine on real-time performance under TSD. Its results confirm the capacity of strategically-timed caffeine administered via chewing gum to disrupt the performance-degrading impact of momentary drowsiness on task performance. The observed dissociation in our caffeine group (compared to placebo) between cognitive test performance and objectively measured drowsiness immediately prior to performing the test, replicates the dissociation pattern previously reported for simulated driving^13^. This dissociation pattern extends our previous findings of caffeine reducing the overall impact of extended wakefulness on cognitive performance by reducing the decline in PVT performance compared to the placebo group^16^, as well as reducing the effect of extended wakefulness on driving errors—both lane keeping and speed maintenance^15^. These findings are consistent with the known alleviating effects of caffeine on gross performance decline over the time course of TSD^4,5,9,19^. We also found that this performance-protective effect was accompanied by reductions in subjective tiredness and irritability without upsetting glucose metabolism and feelings of hunger^14^. However, real-time drowsiness dynamics is a more immediate cause of cognitive failure, and while it correlates with time-since-wake, it is distinct from it—e.g., monitoring driver drowsiness is more predictive of accident risk than timing the total driving time^20^. By adding a near-real-time objective measure of drowsiness (JDS scores, generated over 60-s epochs from infra-red oculography), we were able to analyse the relationship between sleepiness and performance directly, without relying on the time course of TSD as a proxy. Extending our previous findings for simulated driving, here we examined whether caffeine can de-couple real-time drowsiness from the dynamic fluctuation of performance on standard cognitive tests of varying complexity, tapping into *vigilance* (PVT task), simple *decision making* (choice reaction task) and *response inhibition* (Go-NoGo task). This new analysis showed that our caffeine and placebo groups differed quite dramatically in how strongly their momentary drowsiness impacted their response velocities and errors (both lapses and commission errors) across the three cognitive tests. While the placebo group replicated the well-established linear relationship between drowsiness and performance decline^20,21^, the caffeine group showed a distinct pattern of dissociation between the two, which is consistent with our previous findings in simulated driving^13^: i.e., higher drowsiness (increasing JDS scores) did not result in a linear decline in cognitive performance, with the exception of simple reaction time on the PVT task. The magnitude of this dissociation appeared to depend on task complexity, with caffeine significantly reducing the impact of momentary fluctuations of drowsiness on executive performance in the Go-NoGo task, while producing similar but weaker trends in the PRT and SRT tasks. This is consistent with previous findings showing performance on simple tasks, such as the PVT, to be highly sensitive to sleep loss, while more complex tasks such as response inhibition and decision making tend to be less affected^23,29,30^. Similarly, caffeine has been shown to improve response speed in the PVT task to a greater extent than its more complex aspect—lapses^37^ in a 77-h TSD. Further, caffeine’s capacity to improve decision-making during sustained wakefulness has been shown to be limited^9^.

Together, these results suggest that, unlike simple tasks, performance on more complex cognitive tasks may not be driven by levels of alertness alone. Alertness monitoring helps in unpacking these causal links, given the dynamics of alertness are not slaved to the time course of TSD. Combining alertness monitoring with real-time performance assessment in multiple tasks has led us to an unexpected discovery that, in addition to the well-established drowsiness-reduction effect, chewing gum-administered caffeine may also mitigate sleep loss-induced cognitive performance decline by reducing the impact of momentarily fluctuating drowsiness on cognitive task performance. This dissociation was stronger for more complex tasks tapping into executive functioning (the Go-NoGo task in the current study and driving in^13^), than for simple procedural decision making (the two-choice reaction task in the current study) and simple reaction tasks (PVT in the current study). The varying magnitude of this dissociation seems worth further investigation, in order to examine its mechanisms and to inform the development of caffeine-based fatigue countermeasures under challenging operational conditions.

## Methods

### Participants

**Figure 5:**
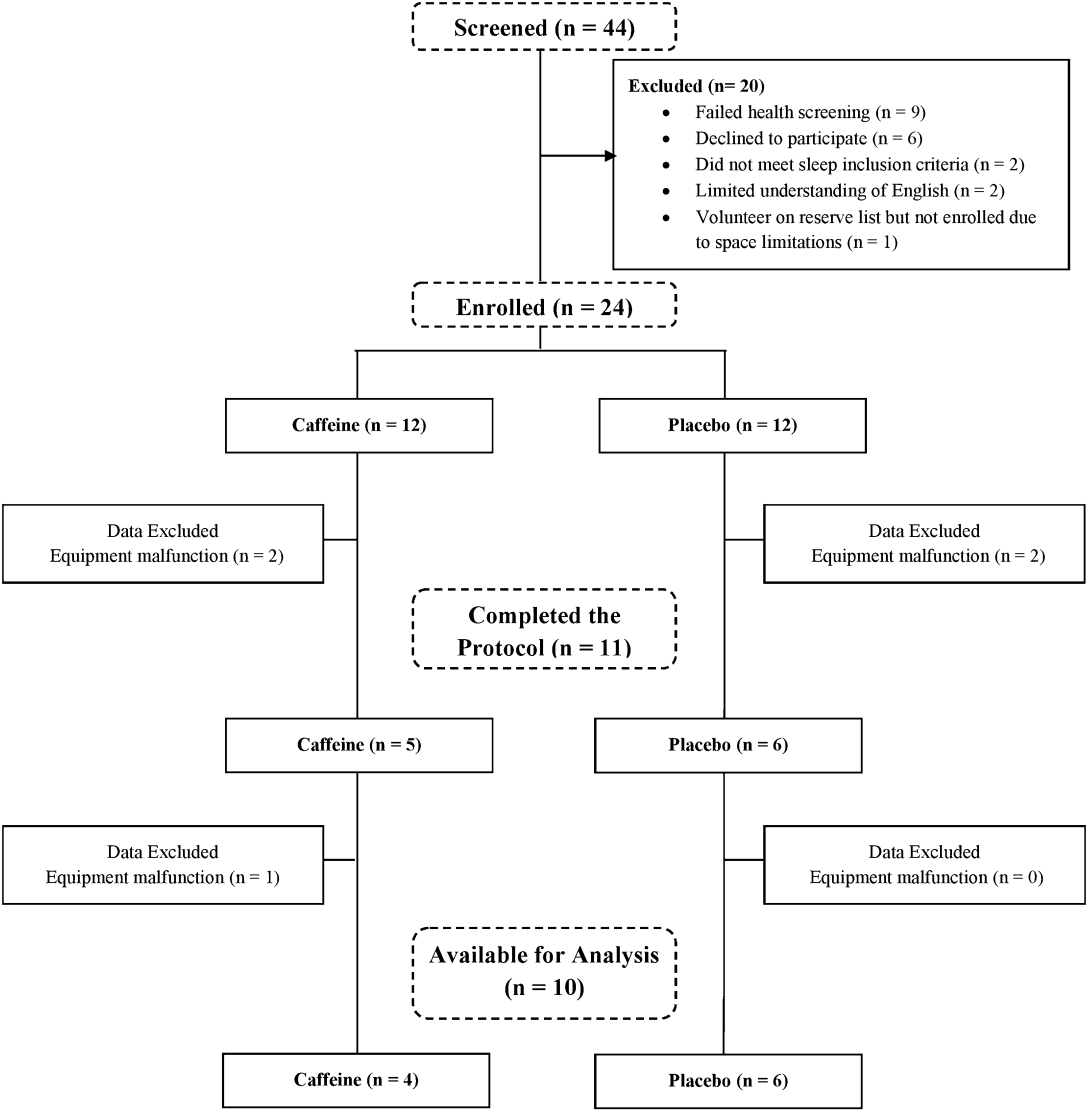
Consort diagram of participant screening, allocation to conditions, protocol completion and the resulting sample used in the current analysis. Adapted from^14^.

The University of South Australia Human Research Ethics Committee approved this study prior to recruiting participants, who were reimbursed for their time. 24 adults (12 male) aged 18–31 years gave written informed consent prior to participating in the study, which was conducted in accordance with Australian National Health and Medical Research Council (NHMRC) guidelines. The inclusion and exclusion criteria are listed in Table 2. The consort diagram for the sample composition for our current analysis is shown in Fig. 5. The resulting sample’s age ranged from 18 to 28 years (M = 22.46 ± 2.73) and BMI ranged from 20.70 to 24.73 kg/m^2^ (M = 21.89 ± 1.37).

**Table 2:** Experimental sample characteristics.

Inclusion criteria	Exclusion criteria
Normal sleep/wake patterns	Smoking
Low or non-consumers of caffeine (< 250 mg daily)	Recreational drug use
BMI below 30	Psychological complaints
	Ill-health (assessed by general health questionnaire and blood toxicology screen)
	Shift-work
	Trans-meridian travel in the last three months

### Procedure

Participants were randomly allocated into either placebo or caffeine group. There were no significant differences in age or BMI between the two groups and gender was evenly distributed between conditions. Participants’ sleep was monitored using sleep diaries and actigraphy for a week prior to the study. They were given a 10-h sleep opportunity. Participants were also required to abstain from caffeine and alcohol, as one week of caffeine abstinence is known to sharpen the effects of its re-introduction and to have minimal withdrawal effects.

**Figure 6:**
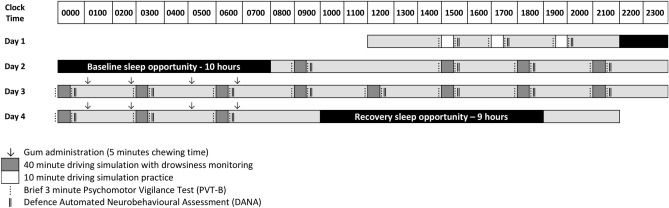
Protocol schematic indicating caffeine administration, simulated driving sessions, cognitive testing sessions and sleep periods.

The experiment took place in the sleep laboratory at the Centre for Sleep Research at the University of South Australia. Ambient temperature was 22 ± 1 °C, light levels were ≤ 50 lx during wakefulness and ≤ 1 lx during sleep. Day 1 of the protocol was dedicated to habituation, training, and oculography equipment calibration. After a 10-h sleep opportunity (2200–0800), data collection commenced at 0800 on Day 2. Participants were constantly monitored and remained awake for 50 h. Every three hours (see Fig. 6) participants drove for 40 min continuously in a medium-fidelity driving simulator (average motion 0.08 m/s^2^ calculated as the root mean square addition of the three motion axes) and controlled by Virtual BattleSpace 2 (VBS-2, Bohemia, USA) to represent a slightly curved two-lane rural highway at dusk. During the driving task, a randomly selected subgroup of eleven participants (five from the caffeine group and six from placebo group) wore an infra-red oculography monitor that quantified drowsiness levels in the form of the Johns Drowsiness Scale (JDS) scores^20,21^.

The JDS scores aggregate oculography parametres over 60-s epochs and range from 0 (very alert) to 10 (very drowsy) with a JDS score between 0 and 4.4 indicating relatively low risk, 4.5–4.9—moderate and scores above 5.0 indicate critical levels of drowsiness^22^. Five consecutive JDS scores immediately preceding the cognitive testing, formed the JDS predictor of cognitive performance.

Directly before and after each driving task, participants completed a 3-min Psychomotor Vigilance Test (PVT-B) and self-reported sleepiness score. Participants also completed a cognitive testing battery using the Defense Automated Neurobehavioral Assessment (DANA) following the post-drive PVT-B.

The caffeine group was administered four doses of caffeine via gum pellets [200 mg/2 pellets per dose, Military Energy Gum (MarketRight INC, Plano, IL, USA)] every two hours (0100, 0300, 0500, 0700) on the first and second nights of sleep deprivation. The placebo group was administered four doses of non-caffeinated chewing gum that was identical in appearance and flavour, at the same time points. All participants were instructed to chew both pellets for a minimum of 5 min, as prior research shows that 85% of the dose is released within 5 min of chewing^19^. The resulting daily dose of 680 mg (85% of 800 mg) is higher than population-wide consumption averages but comparable with typical consumption levels in shift-worker populations^31^. It is also consistent with recent review findings of no adverse neuro-behavioural effects of up to 600 mg/day caffeine consumption^32^. A double-blind randomisation protocol was maintained throughout: all gum labelling was removed and neither the participants nor the researchers knew which group they belonged to.

#### Infra-red oculography: the johns drowsiness scale

Driver drowsiness levels were monitored continuously throughout the simulated drives using the Optalert Alertness Monitoring System (OAMS; OptAlert, Melbourne, VIC, Australia). The OAMS uses spectacle frame-mounted infra-red sensor to continuously monitor eye and eyelid movements during blinks—including their timing, duration, and velocity. These ocular parameters are then combined to quantify drowsiness levels in the form of the Johns Drowsiness Scale (JDS) scores^20^. The OAMS estimates drowsiness by generating a JDS score over regular epochs. Epoch duration was set at 60 s, producing 40 data points for each drive period. For the current analysis we utilised the last five of these 40 data points, covering the 5-min period immediately prior to the cognitive testing. The JDS scores range from 0 (very alert) to 10 (very drowsy) with a JDS score between 0 and 4.4 (inclusive) indicating a relatively low risk level of drowsiness. A score of 4.5–4.9 (inclusive) reflects a moderate drowsiness risk and a score above 5.0 indicates a critically high level of drowsiness risk. The JDS has an established test–retest reliability across different levels of drowsiness (*r* = 0.80;^21^). Its validity evidenced by significant correlations with homeostatic sleep pressure and circadian rhythmicity^22^.

#### Cognitive performance

Psychomotor Vigilance Test (PVT-B). A brief 3 min Psychomotor Vigilance Test (PVT-B)^23^ was presented with a 10 point sleepiness Likert scale. The PVT-B is a shorter version of the traditional 10 min Psychomotor Vigilance Test, which was performed on a hand-held device (PVT-192; Ambulatory Monitoring Inc., Ardsley, NY, USA). The shorter version has been validated by a number of studies as an acceptable substitution for the 10 min version^23–25^. Participants were required to respond as quickly as possible (while avoiding false starts) to a visual stimulus by pressing a button on the hand-held device. Stimuli were presented at random intervals fluctuating between 1 and 4 s. The performance measures for the PVT-B were the number of lapses (defined as reaction time > 355 ms) and the average of the fastest 10% of responses.

Single-item sleepiness self-rating: participants were asked to rate how sleepy they felt on a Likert scale format from 1 (not sleepy) to 10 (sleepy) and enter their ratings on the PVT-B device.

Defense Automated Neuropsychological Assessment (DANA) battery. The DANA unit is a small, hardened, handheld device^26^ loaded with a range cognitive tests, from which three were selected—simple reaction time (SRT), procedural reaction time (PRT) and Go-NoGo (GNG) tasks. It has been shown to have good reliability and internal validity, comparable to those reported for the corresponding subtests in the NeuroCognitive Assessment Tool and the Automated Neuropsychological Assessment Metrics^26–28^. Participants responded to stimuli by tapping the screen with a handheld stylus. The following DANA tests were utilised in the current study.

##### Simple reaction time (SRT) task

This task requires the participant to tap on the location of the yellow bulls-eye symbol as quickly as possible each time it appeared on the DANA screen. The task contained 40 trials with stimulus presentation time of 900 ms and inter-stimulus interval ranging from 600 to 3000 ms.

##### Procedural reaction time (PRT) task

The screen displays one of four single-digit numbers, and participants are asked to respond by tapping the left button if the number was small (2 or 3) or the right button if the number was large (4 or 5). The task contained 32 trials. Maximum stimulus presentation time was 2000 ms and inter-stimulus interval fluctuated from 500 to 1000 ms.

##### Go-Nogo (GNG) task

This is a response inhibition task requiring to fire at the target (white silhouette) in the window of a building sketched on the screen by tapping the ‘fire’ button, and to withhold their response when a non-target (green silhouette) appeared in the same window. The task contained 30 trials. Maximum stimulus presentation time was 1500 ms, with the inter-stimulus interval fluctuating from 1000 to 1750 ms.

### Statistical analyses

All data analyses were conducted within R environment, version 3.5.0^33^. TIDYVERSE package version 1.2.1^34^ and COWPLOT package, version 0.9.4^35^ were used for data organisation and visualisations. Main effects of caffeine and drowsiness, as well as their interactions were estimated with Satterthwaite approximated *t* statistics derived from Linear Mixed-effects Modelling analyses conducted with the lme4 package^36^. Mixed-effects modelling takes advantage of the multiple repeat measurements in our protocol, which compensated for the relatively low number of participants in our caffeine (n = 4) and placebo (n = 6) groups, producing over 60 observations per condition. The resulting degrees of freedom reported in Table 1 reflect the actual statistical power of our analyses: these *df* values range from 35.6 to 94.9 (M = 74.7) and indicate that our analyses are adequately powered.

## Supplementary Information

Supplementary Information

## Data Availability

All data is available in the main text and the supplementary materials.

## Author note

Material has been reviewed by the Walter Reed Army Institute of Research. There is no objection to its presentation and/or publication. The opinions or assertions contained herein are the private views of the author, and are not to be construed as official, or as reflecting true views of the Department of the Army or the Department of Defense. The investigators have adhered to the policies for protection of human subjects as prescribed in AR 70–25. We thank Prof Gary Wittert for his support with participant recruitment.
